# Supplementary material for: Mapping the evolution of fertility support policies in China: A content and instrumental analysis
Source: PLoS One. 2025 Oct 9;20(10):e0332137. doi: 10.1371/journal.pone.0332137 (PMC12510515; doi:10.1371/journal.pone.0332137)
Supplement: S1 Appendix — (ZIP) [file pone.0332137.s001.zip › S1 Appendix. 226 original policy documents/101-全国残疾人康复工作办公室关于印发《儿童残疾预防“十二五”实施方案》实施办法的通知(FBM-CLI-4-170544).doc]

全国残疾人康复工作办公室关于印发《儿童残疾预防“十二五”实施方案》实施办法的通知

**发布部门：** [全国残疾人康复工作办公室](https://www.pkulaw.com/law/chl?Aggs.IssueDepartment=7060455)

**发文字号：**全康办〔2012〕8号

**发布日期：**2012.02.13

**实施日期：**2012.02.13

**时效性：** [现行有效](https://www.pkulaw.com/law/chl?Aggs.TimelinessDic=01)

**效力级别：** [部门规范性文件](https://www.pkulaw.com/law/chl?Aggs.EffectivenessDic=XE0302)

**法规类别：** [老少妇幼残保护](https://www.pkulaw.com/law/chl?Aggs.Category=01604)

全国残疾人康复工作办公室关于印发《儿童残疾预防“十二五”实施方案》实施办法的通知
（全康办〔2012〕8号）

各省、自治区、直辖市残疾人康复工作办公室，新疆生产建设兵团残联，黑龙江农垦总局残联：
　　为贯彻落实《儿童残疾预防“十二五”实施方案》提出的任务要求，全国残疾人康复工作办公室制定了《〈儿童残疾预防“十二五”实施方案〉实施办法》，现印发给你们，请结合本地实际，认真组织实施。

全国残疾人康复工作办公室
二○一二年二月十三日

　　《儿童残疾预防“十二五”实施方案》实施办法

　　第二次全国残疾人抽样调查显示，全国约有0-6岁残疾儿童167.8万。我国每年出生缺陷儿童总数高达80-120万，新增0-6岁残疾儿童19.9万。出生缺陷和儿童残疾给家庭和社会造成沉重的精神和经济负担，成为突出的公共卫生问题和社会问题。
　　积极开展儿童残疾预防与康复工作，宣传残疾预防和早期康复知识，对于减少出生缺陷、提高人口素质具有重要意义。早发现、早干预、早康复是残疾儿童康复的关键，儿童越早得到甄别、越及时地进行治疗和康复，康复的效果就越好，是一项投入少、见效快、惠及残疾儿童一生的抢救性工程。
　　为加强儿童残疾预防工作，根据《儿童残疾预防“十二五”实施方案》，特制定本实施办法。
　　**一、**任务目标
　　在全国31个城市开展残疾儿童随报及早期康复工作试点，探索建立早预防、早筛查、早转介、早治疗、早康复的工作机制。
　　在全国开展儿童残疾预防和早期康复宣传教育活动，到2015年，儿童残疾预防与早期康复知识的知晓率达到80%。

　　**二、**工作进度
　　（一）2012年，确定开展残疾儿童随报及早期康复工作试点城市。各省制定工作方案，启动残疾儿童随报及早期康复工作试点工作。
　　（二）2013年，组织业务培训，指导各试点城市开展儿童残疾预防工作。广泛开展儿童残疾预防和早期康复宣传教育活动。进行中期评估。
　　（三）2014年，召开残疾儿童随报及早期康复工作试点工作经验交流会议，总结试点经验。

　　**三、**主要措施
　　（一）健全和完善儿童残疾预防工作体系。
　　1. 组织管理网络。
　　建立儿童残疾预防政府主导、相关部门各尽其职、协调运作的工作机制。试点城市在政府统一领导下，卫生、民政、人口计生、残联等部门共同制定本地区儿童残疾预防工作方案，并组织实施。
　　试点城市政府负责出台残疾儿童筛查和早期康复相关优惠政策，建立长效的经费保障机制，完善和落实有利于残疾儿童筛查、转介和康复的相关法规和政策，保障残疾儿童接受康复服务的权利。
　　--卫生部门负责组织婚前卫生指导、咨询和医学检查，做好新婚夫妇、孕产期妇女保健工作，早期发现出生缺陷儿并及时进行医学干预和治疗，将残疾儿童筛查工作纳入国家基本公共卫生服务规范0-36个月儿童健康管理和0-6岁儿童健康体检，对各级医务人员开展相关知识和技能培训，及时发现疑似残疾儿童，并到定点医疗机构进行确诊，将确诊的残疾儿童信息与残联共享。
　　--民政部门负责利用婚姻登记管理平台，做好儿童残疾预防和早期康复知识宣传教育，引导群众自觉参加婚前卫生指导和医学检查，制定优惠政策，对各类贫困残疾儿童进行康复医疗救助。
　　--人口计生部门负责利用人口计生工作网络组织开展优生优育宣传活动，普及残疾预防知识。
　　--残联负责制定相关技术标准，规范各类残疾儿童康复机构建设，提供康复机构信息的咨询服务，将确诊的残疾儿童及时转介至相应康复机构进行康复安置。
　　2.技术指导网络。
　　全国残疾人康复工作办公室成立专家技术指导组，制定相关技术标准，指导各试点城市开展儿童残疾预防工作，进行检查评估。
　　试点城市残疾人康复工作办公室成立专家技术指导组，负责对基层社区医务人员、妇幼保健人员进行培训，使他们掌握基本的残疾儿童筛查技术和方法，熟悉残疾儿童筛查工作流程，对定点医疗机构医生进行残疾诊断相关技术培训，指导制定治疗和康复方案。
　　3.工作流程。
　　（二）加强人员培训，建立工作队伍。
　　加大基层儿童保健医务工作者培训力度，使他们掌握基本的筛查技术和方法，熟悉儿童残疾筛查工作流程，对专科医院医生进行残疾诊断技术培训和康复知识培训。注重残联系统残疾儿童筛查诊断队伍的培养，加强人员培训，保证工作质量。
　　全国残疾人康复工作办公室举办国家级培训班，依托全国专家技术指导组，为31个试点城市培训儿童残疾预防管理和技术骨干。
　　（三）建立残疾儿童随报及早期康复试点制度。
　　城市社区卫生服务中心和乡镇卫生院等基层卫生服务机构负责开展0-6岁残疾儿童早期筛查工作，将发现的疑似残疾儿童在县（市、区）妇幼保健机构备案，并转介至市（地）定点医疗机构进行确诊。
　　定点医疗机构负责对疑似残疾儿童进行确诊，建立残疾儿童信息数据库，将有医疗需求的残疾儿童转介至相关医疗机构进行诊治，将有康复需求的残疾儿童信息转介至残联。
　　残联负责将有康复需求的残疾儿童转介至相应康复机构接受早期康复，促进功能改善，减轻残疾程度，进行康复安置。
　　探索建立卫生和残联相互协作残疾儿童数据库，通过儿童保健网络对残疾儿童进行筛查，将可疑残疾儿童相关数据录入数据库，残疾诊断机构将残疾儿童的诊断情况录入数据库，残疾治疗和康复机构将接受治疗或康复的儿童情况录入数据库，实现对儿童残疾的常规监测。
　　残疾儿童随报及早期康复工作试点城市要符合以下条件：
　　组织管理网络健全。试点城市要在政府统一领导下，卫生、民政、人口计生、残联等部门协作配合，特别是残联要与卫生部门密切联系，在现有的新生儿筛查和儿童保健服务内容中明确增加儿童残疾筛查内容；技术指导网络健全。试点城市妇幼保健网络健全，具备残疾儿童筛查、诊断技术资源、能够通过基层妇幼保健网络开展残疾儿童早期筛查；筛查诊断转介网络健全。试点城市要有建立残疾儿童筛查、诊断、转介制度的基础，健全残疾儿童筛查、诊断工作流程，能将发现的残疾儿童及时转介至相应医疗和康复机构；康复服务网络健全。试点城市有各类残疾儿童康复机构，能够及时为转介出的残疾儿童提供康复服务；经费保障。试点城市要投入相应配套经费用于残疾儿童随报及早期康复工作。
　　各地要做好试点城市的遴选工作，选择有工作基础、积极性高的城市，特别是卫生、残联合作关系较好的地区；加强与卫生、人口计生部门，特别是卫生部门的合作，出台保障儿童残疾预防制度性文件和政策，建立儿童残疾预防工作机制；纳入公共卫生项目，争取财政支持，建立稳定儿童残疾预防经费保障机制；及时总结、推广试点地区工作经验，从残疾人事业发展的长远角度，统筹规划，不局限于试点地区，逐步在全省范围建立残疾儿童筛查随报制度。
　　（四）宣传儿童残疾预防与早期康复知识。
　　针对新婚夫妇、孕产期妇女、0-6岁儿童等重点人群，有计划、有重点地普及儿童残疾预防和早期康复知识，广泛开展健康教育、婚前卫生指导、咨询和医学检查、孕前和孕早期医疗保健，宣传优生优育知识，避免接触有毒有害物质，减少出生缺陷。
　　在妇幼保健院（站）、社区卫生服务中心（站）、乡镇卫生院、村卫生室、社区康复站、残疾人活动场所等，宣传残疾儿童预防和早期康复知识。
　　组织儿童残疾预防宣传活动，利用“全国助残日”、“全国爱耳日”、“世界孤独症日”、“精神卫生日”等节日，举办形式多样的宣传活动，印制宣传画册、宣传品、宣传展板等，宣传健康的生活方式，普及安全生产、职业病防治、精神卫生等残疾预防知识。
　　开展宣传教育活动要根据地区、对象、目的、内容，科学设计、综合合理运用适宜的方法，要充分调动社区群众广泛参与。宣传教育方法可采取多种方法，如语言教育方法：口头交谈、健康咨询、专题讲座、小组座谈和大会报告、演讲等；文字教育方法 ：标语、传单、小册子、折页、报刊、墙报、专栏、宣传画；形象化教育方法：图片、照片、标本、模型、示范、演示等；电化教育方法：广播、电视、电影以及投影、幻灯、VCD、录音带、录像带等。

　　**四、**经费保障
　　中央财政对承担“十二五”残疾儿童随报及早期康复工作试点城市给予补贴，2012至2015年，中央财政支持每个试点城市30万元。地方财政按不低于中央财政补贴标准投入相应配套经费。

　　**五、**检查评估
　　各级残疾人康复工作办公室要按照《儿童残疾预防“十二五”实施方案》的要求，根据任务完成情况，定期进行检查评估。
　　全国残疾人康复工作办公室于2013年进行儿童残疾预防工作中期检查，2015年进行全面检查验收。
　　附件：
　　1.残疾儿童随报及早期康复工作任务分配表
　　2.“十二五”残疾儿童筛查、转介、康复登记表
　　3.“十二五”儿童残疾预防工作汇总表
　　附件1：残疾儿童随报及早期康复工作任务分配表

| 地区 | 试点城市 |
| --- | --- |
| 北京 | 1 |
| 天津 | 1 |
| 河北 | 1 |
| 山西 | 1 |
| 内蒙古 | 1 |
| 辽宁 | 1 |
| 吉林 | 1 |
| 黑龙江 | 1 |
| 上海 | 1 |
| 江苏 | 1 |
| 浙江 | 1 |
| 安徽 | 1 |
| 福建 | 1 |
| 江西 | 1 |
| 山东 | 1 |
| 河南 | 1 |
| 湖北 | 1 |
| 湖南 | 1 |
| 广东 | 1 |
| 广西 | 1 |
| 海南 | 1 |
| 重庆 | 1 |
| 四川 | 1 |
| 贵州 | 1 |
| 云南 | 1 |
| 西藏 | 1 |
| 陕西 | 1 |
| 甘肃 | 1 |
| 青海 | 1 |
| 宁夏 | 1 |
| 新疆 | 1 |
| 合计 | 31 |

　　附件2：“十二五”残疾儿童筛查、转介、康复登记表
　　填表单位（公章）：
　　填表人：　　　　　　　　　　　　　 审核人：　　　　　　　填表日期：

| 儿童姓名 |  | | 性别 | | | |  |
| --- | --- | --- | --- | --- | --- | --- | --- |
| 出生日期 | 年 月 日 | | 民族 | | | |  |
| 家长姓名 |  | | 身份证号 | | | |  |
| 联系方式 | 宅电 |  | 手机 | | | |  |
| 家庭经济状况 | □家庭人均收入低于当地城乡居民最低生活保障  □农村领取社会救助金  □家庭经济困难 | | | | | | |
| 通讯地址 |  | | | 邮政编码 | | |  |
| 筛查情况 | 筛查机构 |  | | | 筛查工具 | |  |
|  | 筛查结果 | □疑似视力残疾 □疑似听力残疾 □疑似言语残疾  □疑似肢体残疾 □疑似智力残疾 □疑似精神残疾 | | | | | |
| 诊断情况 | 诊断机构 |  | | | | 诊断工具 |  |
|  | 诊断结果 | □视力残疾 □听力残疾 □言语残疾  □肢体残疾 □智力残疾 □精神残疾 | | | | | |
| 转介情况 | 是否转介 | □是 □否 | | | | | |
|  | 转介机构 |  | | | | | |

　　填表说明：此表由基层卫生人员填写，一式两份，1份存档，1份上报至县残联，由县残联逐级上报至省（自治区、直辖市）残联。
　　附件3：“十二五”儿童残疾预防工作汇总表
　　填报单位（公章）：

| 0-6岁儿童数 | ＿＿＿人 | | |
| --- | --- | --- | --- |
| 筛查0-6岁儿童数 | ＿＿人 | 疑似残疾  儿童数 | ＿＿人 |
| 诊断0-6岁  残疾儿童数 | ＿＿人，其中，视力残疾儿童＿人，听力残疾儿童＿人，言语残疾儿童＿人，肢体残疾儿童＿人，智力残疾儿童＿人，精神残疾儿童＿人 | | |
| 培训情况 | 培训基层残疾儿童筛查工作人员＿＿人，培训基层残疾儿童诊断工作人员＿＿人，培训基层残疾儿童管理工作人员＿＿人 | | |
| 宣传教育情况 | 开展儿童残疾预防和早期康复宣传教育活动＿＿次，  接受儿童残疾预防和早期康复宣传教育人数＿＿人 | | |
| 知晓率情况 | 医务人员对儿童残疾预防与早期康复知识知晓率＿%，重点人群对儿童残疾预防与早期康复知识的知晓率＿% | | |

　　填表人：　　　　　　　　　　　　　　审核人：
　　填表日期：
　　填表说明：
　　此表由县残联填写，逐级汇总上报至省（自治区、直辖市）残联，由省残联备案审核汇总后，报中国残联信息中心。

© 北大法宝：（[www.pkulaw.com](https://www.pkulaw.com/)) 专业提供法律信息、法学知识和法律软件领域各类解决方案。北大法宝为您提供丰富的参考资料，正式引用法规条文时请与标准文本核对。
欢迎查看所有[产品和服务](https://www.pkulaw.com/helps/2.html)。法宝快讯：[如何快速找到您需要的检索结果？](https://www.pkulaw.com/helps/70.html)[法宝](https://www.pkulaw.com/helps/69.html)V6有何新特色？

扫描二维码阅读原文

**原文链接：**<https://www.pkulaw.com/chl/6d3b45e60a607646bdfb.html>
